# Discrepancies between fall risk and fall awareness in hospitalized elderly patients with cataracts: a cross-sectional study

**DOI:** 10.3389/fmed.2026.1851395

**Published:** 2026-06-10

**Authors:** Jinxiu Yao, Danling Fang, Pei Xu, Shijie Liu, Chunxiu Zhou, Lifei Su, Danhong Kang, Xiaochen Su, Lili Ma, Minling Mo

**Affiliations:** 1 Department of Nursing, Shanghai Sixth People’s Hospital Affiliated to Jiao Tong University, School of Medicine, Shanghai, China; 2 Department of Nursing, Shanghai University of Medicine and Health Sciences Affiliated Zhoupu Hospital, Shanghai, China; 3 School of Medicine, Tongji University, Shanghai, China; 4 Department of Nursing, Shanghai East Hospital, School of Medicine, Tongji University, Shanghai, China; 5 Department of Nursing, Shanghai Sixth People’s Hospital Fujian, Fujian, China

**Keywords:** cataract, elderly, fall awareness, fall risk, inpatients

## Abstract

**Objective:**

To investigate the discrepancies between fall risk and fall awareness among hospitalized elderly cataract patients, providing a basis for implementing precise fall prevention assessments and interventions in clinical practice.

**Methods:**

Using convenience sampling, 526 elderly cataract patients hospitalized in a tertiary hospital in Shanghai from March 2025 to September 2025 were selected as study subjects. A cross-sectional survey was conducted using a general information questionnaire, the Johns Hopkins Fall Risk Assessment Scale, and the Fall Awareness Scale to analyze the distribution characteristics and differences between fall risk and fall awareness, and to compare the differences in their influencing factors.

**Results:**

The fall risk score of hospitalized elderly cataract patients was (6.56 ± 3.44), indicating a moderate level, while the fall awareness score was (54.45 ± 11.27), also indicating a moderate level. There was no significant difference (*χ*^2^ = 3.074, *p* > 0.05) in fall awareness scores among hospitalized elderly cataract patients with different fall risk levels. In the high-risk group, 62.50% (25/40) of patients exhibited insufficient awareness; in the medium-risk group, 35.20% (107/304) showed insufficient awareness, while 27.96% (85/304) exhibited excessive awareness; in the low-risk group, 67.58% (123/182) displayed excessive awareness. Factors associated with fall risk included age, marital status, education level, sleep quality, residence, medication history, underlying diseases, etc.

**Conclusion:**

This study clearly demonstrates the widespread risk-awareness mismatch among hospitalized elderly cataract patients. Hospitalized elderly cataract patients exhibit a moderate level of fall risk but widespread fall awareness deviations, characterized by insufficient awareness among high-risk patients coexisting with excessive awareness among low-risk patients. Simple assessment of objective fall risk fails to identify patients’ subjective cognitive biases. It is recommended to incorporate fall awareness assessment into routine nursing screenings to implement individualized and precise fall prevention interventions.

## 1 Introduction

Falls are common accidental events among elderly people, referring to sudden, involuntary, and unintentional changes in body position that result in the individual landing on the ground or a lower surface (1). Impaired visual function is a significant risk factor for falls in the elderly, with cataracts—one of the most prevalent vision-impairing conditions globally—severely affecting their ability to walk safely and recognize environmental hazards. According to World Health Organization data (2), approximately 94 million people worldwide are affected by cataracts, with over 80% of cases occurring in individuals aged 60 and above (2). As the population ages, the number of elderly cataract patients in China continues to rise annually, with projections exceeding 120 million by 2030 (3). Consequently, fall-related health issues are becoming increasingly prominent.

Cataracts can lead to blurred vision, decreased contrast sensitivity, and loss of stereoscopic vision, severely impairing patients’ balance and environmental perception (4), increasing their risk of falls by 2–3 times compared to ordinary elderly people (5). Studies indicate that cataract-related visual impairment is associated with an increased risk of falls, and cataract surgery can reduce the frequency of falls in elderly people (6, 7). Domestic research also suggests that the visual function level of age-related cataract patients is significantly correlated with fall-related factors (such as balance and mobility), indicating that this group exhibits fall-prone characteristics (8). Once a fall occurs, it may lead to severe complications such as fractures and craniocerebral injury (9), exacerbating physical dysfunction and even resulting in disability. A study indicates that visual impairment is a significant risk factor for falls. Besides the heightened risk of falls linked to various visual disorders, the study also discovered an increased risk of fractures. Elderly cataract patients face a 28% higher risk of fractures compared to their peers with good vision, which significantly reduces their quality of life and increases the burden of family care (10). Although fall risk assessment scales have been widely adopted in clinical practice to screen high-risk populations, with stratified preventive measures implemented based on risk levels, existing evaluation tools predominantly focus on the identification and stratification of objective risk factors, paying less attention to patients’ subjective perception of their own fall risk and alertness state (11). Fall awareness refers to an individual’s ability to recognize potentially relevant and unpredictable changes in the environment, reflecting the brain’s vigilance toward fall incidents, which encompasses three characteristics: subjective risk perception, behavioral execution intention, and fall context (12). Domestic research has completed the translation and validation of the “Fall Awareness Scale,” providing a foundational tool for clinically assessing patients’ risk perception (13). However, for hospitalized elderly cataract patients, whether their objective fall risk aligns with subjective fall awareness, the distribution characteristics of any discrepancies, and whether the influencing factors are consistent remain lacking systematic empirical evidence.

This study focuses on elderly cataract patients, employing a cross-sectional survey to systematically evaluate the current status of their fall risk and fall awareness, and conducts an in-depth analysis of the differences between the two as well as the characteristics and influencing factors. The aim is to provide an evidence-based foundation to establish a collaborative assessment model of “objective risk—subjective awareness” and to develop individualized fall prevention strategies, thereby enhancing the precision of inpatient safety and fall prevention interventions for elderly cataract patients.

## 2 Materials and methods

### 2.1 Study design and setting

Using a convenience sampling method, elderly cataract patients hospitalized in two campuses of a tertiary hospital in Shanghai were selected as the study subjects from March 2025 to September 2025. Inclusion criteria: ① Age ≥ 60 years; ② Meeting the clinical diagnostic criteria for cataract and being treated as inpatients at this hospital; ③ Having good communication skills, being able to understand the study content and complete questionnaires independently; ④ Written informed consent was obtained from all participants and their family members prior to enrollment. Exclusion criteria: ① Individuals with significant motor, sensory, or balance dysfunction due to disease; ② Comorbid severe mental or psychological disorders; ③ Presence of vestibular dysfunction disorders such as otogenic vertigo. Sample size estimation: For survey - based studies, the sample size should be 5–10 times the number of items. This study included 18 items in the general information questionnaire, 7 items in the Johns Hopkins Fall Risk Assessment Scale, and 21 items in the Fall Awareness Scale, totaling 48 items, the initial sample size was 240–480. Considering a 20% sample attrition rate, the calculated sample size ranged from 300 to 600 cases. This study ultimately collected 526 valid questionnaires, meeting the sample size requirements. The study was approved by the hospital ethics committee (Ethics No. JJSYYLL - 2024 – 068, approved on November 1, 2024), and all participants and their family members provided informed consent and voluntarily participated in the study.

### 2.2 Measures

#### 2.2.1 General information questionnaire

Referring to similar literature (14) and based on the implementation foundation of this study, a self-designed questionnaire was created. All questionnaires were administered within 24 h after admission to avoid perioperative psychological fluctuations that may affect fall alertness scores. It includes gender, age, marital status, occupation, education level, smoking, alcohol consumption, sleep quality, residence, activity, payment method, medication history, surgical history, underlying diseases, self-care ability, vision, hearing, and history of falls in the past 6 months. Visual function was assessed using the standard international logarithmic visual acuity chart, and the best-corrected visual acuity was recorded in logMAR units, then graded into mild, moderate, and severe impairment. Surgical history was dichotomized as “yes/no” in univariate analysis. For better clinical interpretation, cataract-related surgery (pre-operative, unilateral post-operative, bilateral post-operative) and non-cataract surgery should be distinguished in further stratified analysis.

#### 2.2.2 Johns Hopkins fall risk assessment tool, JHFRAT

The Johns Hopkins Fall Risk Assessment Tool (JHFRAT), published in 2005, is used for predicting in - hospital fall risk (15). This scale was translated into Chinese by Zhang et al. in China (16). The tool comprises two parts: ① Directly classifying patients’ fall risk based on four scenarios outlined in the criteria; ② Risk stratification based on total scores: <6 points indicates low risk, 6–13 points indicates moderate risk, and >13 points indicates high risk. The Chinese version of the Johns Hopkins Fall Risk Assessment Tool shows an overall Cronbach’s *α* coefficient of 0.791, an inter - rater reliability of r = 0.949, and exploratory factor analysis extracted four common factors with a cumulative variance contribution rate of 62.437%. In conclusion, this scale is suitable for assessing fall risk among hospitalized patients in China. In the present study, the Cronbach’s *α* coefficient of the JHFRAT in this sample was 0.792.

#### 2.2.3 Self-awareness of falls in elderly, SAFE

This scale was developed by Shyu et al. (13) and was subjected to reliability and validity testing among hospitalized elderly patients in Taiwan, with a Cronbach’s *α* coefficient of 0.810. It aims to address the lack of attention to patients’ subjective cognition in traditional objective fall risk assessment tools (such as the Morse Scale) and is used to evaluate hospitalized elderly patients’ subjective awareness of fall risk. The scale consists of four dimensions: activity safety and environmental awareness, physical function awareness, medication awareness, and cognitive - behavioral awareness, with a total of 21 items. It uses a Likert 5 - point scoring system (1 = strongly agree, 5 = strongly disagree), where higher total scores indicate greater fall awareness. Domestic studies have shown the scale’s good reliability and validity. Hu et al. verified its overall Cronbach’s *α* as 0.943 and test - retest reliability as 0.900 among elderly individuals in nursing institutions (17). They extracted four common factors through exploratory factor analysis with a cumulative variance contribution rate of 74.609% and proposed an optimal cutoff value of 54 points. He et al. confirmed its overall Cronbach’s *α* as 0.923 among hospitalized elderly patients (18). In summary, the SAFE scale can assess fall awareness levels in elderly individuals across physical function, environmental safety, medication, and cognitive - behavioral aspects, making it suitable for nursing institutions and hospitalized elderly populations in China. In the present study, the overall Cronbach’s *α* of the SAFE scale was 0.942. In this study, sample tertiles were used to categorize fall awareness levels.

#### 2.2.4 Criteria for defining fall risk–alertness mismatch

Fall risk (JHFRAT) was categorized into low/moderate/high, and fall awareness (SAFE) was categorized into low/moderate/high. Insufficient fall alertness refers to low or moderate alertness in high-risk patients or low alertness in moderate-risk patients. Excessive fall alertness refers to moderate or high alertness in low-risk patients or high alertness in moderate-risk patients. The division of fall risk level was consistent with fall alertness level.

### 2.3 Data collection

This study adhered to the inclusion and exclusion criteria for selecting participants. Two investigators, after receiving standardized training, collected data from March 2025 to September 2025. The study purpose was explained to participants through face-to-face interviews. Questionnaires were administered after written informed consent was obtained from all participants and their families, with clear instructions provided on completion methods and precautions. Participants were instructed to answer based on their actual thoughts, and objective explanations were given for any unclear items. Questionnaires were collected on-site and individually reviewed to ensure quality. Ultimately, 526 valid copies each of the general information questionnaire, Johns Hopkins Fall Risk Assessment Tool, and Fall Awareness Scale were recovered, achieving a 100% valid questionnaire return rate.

### 2.4 Statistical analysis

IBM SPSS Statistics 25.0 was used for data analysis. Count data were analyzed using frequencies and percentages (%), chi-square test (*χ*^2^) was used; Fisher’s exact test was used for cells with expected frequency < 5. Measurement data with normal distribution were presented as mean ± standard deviation (x̄ ± s), and measurement data with non - normal distribution were expressed as M (P25, P75). For non - normally distributed measurement data, the Mann - Whitney U test was used for comparisons between two groups, while the Kruskal - Wallis H rank - sum test was employed for comparisons among three or more groups. For significant Kruskal-Wallis tests, Dunn’s *Post-hoc* test with Benjamini-Hochberg adjustment was used for pairwise comparisons between groups. A *p* < 0.05 was considered statistically significant.

## 3 Results

### 3.1 General characteristics

This study collected 526 valid questionnaires, including 202 male cases (38.4%) and 324 female cases (61.6%). Among the participants, 248 cases (47.15%) were aged 60–69 years, 249 cases (47.34%) were aged 70–79 years, and 29 cases (5.51%) were aged 80 years or older. Other general demographic data are presented in Table 1.

**Table 1:** General characteristics (*n =* 526).

Variables	Number	Percentage
Gender		
Male	202	38.40%
Female	324	61.60%
Age		
60–69	248	47.15%
70–79	249	47.34%
≥80	29	5.51%
Marital status		
Single	20	3.80%
Married	433	82.32%
Divorced	27	5.13%
Bereft spouse	46	8.75%
Occupation		
Peasant	154	29.28%
Worker	242	46.01%
Public servant	39	7.41%
Professionals	42	7.98%
Liberal professions	49	9.32%
Educational level		
Primary school and below	199	37.83%
Junior middle school	208	39.54%
High school or above	119	22.62%
Smoking		
No	464	88.21%
Yes	62	11.79%
Drinking		
No	506	96.20%
Yes	20	3.80%
Sleep quality		
Good	248	47.15%
General	233	44.30%
Bad	45	8.56%
Residence		
Living alone	37	7.03%
Living with other	489	92.97%
Activity		
Independent activities	498	94.68%
Assistance needed	28	5.32%
Payment		
Medical insurance	462	87.83%
Self paid	64	12.17%
Medication history		
No	115	21.86%
Yes	411	78.14%
Surgical history		
No	181	34.41%
Yes	345	65.59%
Number of underlying diseases		
0	68	12.93%
1	211	40.11%
2	195	37.07%
3 or more	52	9.89%
Self-care ability		
Complete self-help	61	11.60%
Mild dependence	396	75.29%
Moderate dependence	36	6.84%
Severe dependence	33	6.27%
Hearing		
Normal	400	76.05%
Abnormal	126	23.95%
Visual impairment		
Mild	156	29.66%
Middle	335	63.69%
Severe	35	6.65%
Falls history in the past 6 months		
No	514	97.72%
Yes	12	2.28%

### 3.2 Fall risk level

Among the 526 participants, according to the risk classification criteria of the Johns Hopkins Fall Risk Assessment Tool, 182 cases (34.60%) were classified as low risk, 304 cases (57.79%) as medium risk, and 40 cases (7.60%) as high risk. Univariate analysis revealed that age, marital status, educational level, sleep quality, residence, medication history, number of underlying diseases, degree of visual impairment, and fall history within the past 6 months showed statistically significant differences (*p* < 0.05) among patients with different fall risk levels, whereas gender, occupation, smoking, alcohol consumption, activity pattern, payment method, surgical history, self - care ability, hearing, height, and weight showed no statistically significant differences (*p* > 0.05) among the different risk level groups. *Post-hoc* Dunn-BH pairwise comparisons revealed statistically significant differences in fall risk levels across subgroups of age, marital status, education level, sleep quality, residence, medication history, number of underlying diseases, degree of visual impairment, and fall history within the past 6 months (*p* < 0.05), in Table 2.

**Table 2:** Comparison of fall risk and fall alertness results (*n =* 526).

Variables	Fall risk assessment						Fall alertness assessment					
	Low-risk	Medium-risk	High-risk	Total	Statistic	*P*-value	Low level	Medium level	High level	Total	Statistic	*P*-value
Gender					4.253	0.119					2.162	0.339
Male	77 (42.31%)	115 (37.83%)	10 (25.0%)	202 (38.4%)			72 (41.14%)	67 (34.36%)	63 (40.38%)	202 (38.4%)		
Female	105 (57.69%)	189 (62.17%)	30 (75.0%)	324 (61.6%)			103 (58.86%)	128 (65.64%)	93 (59.62%)	324 (61.6%)		
Age					37.535	<0.001					30.700	<0.001
60–69	106 (58.24%)	132 (43.42%)	10 (25.0%)	248 (47.15%)			107 (61.14%)	87 (44.62%)	54 (34.62%)	248 (47.15%)		
70–79	71 (39.01%)	157 (51.64%)	21 (52.5%)	249 (47.34%)			61 (34.86%)	102 (52.31%)	86 (55.13%)	249 (47.34%)		
≥80	5 (2.75%)	15 (4.93%)	9 (22.5%)	29 (5.51%)			7 (4.0%)	6 (3.08%)	16 (10.26%)	29 (5.51%)		
Marital status					17.333	0.008					22.135	0.001
Single	10 (5.49%)	9 (2.96%)	1 (2.5%)	20 (3.8%)			6 (3.43%)	9 (4.62%)	5 (3.21%)	20 (3.8%)		
Married	156 (85.71%)	251 (82.57%)	26 (65.0%)	433 (82.32%)			151 (86.29%)	169 (86.67%)	113 (72.44%)	433 (82.32%)		
Divorced	6 (3.3%)	16 (5.26%)	5 (12.5%)	27 (5.13%)			8 (4.57%)	7 (3.59%)	12 (7.69%)	27 (5.13%)		
Bereft spouse	10 (5.49%)	28 (9.21%)	8 (20.0%)	46 (8.75%)			10 (5.71%)	10 (5.13%)	26 (16.67%)	46 (8.75%)		
Occupation					4.517	0.808					11.597	0.170
Peasant	52 (28.57%)	87 (28.62%)	15 (37.5%)	154 (29.28%)			53 (30.29%)	60 (30.77%)	41 (26.28%)	154 (29.28%)		
Worker	83 (45.6%)	140 (46.05%)	19 (47.5%)	242 (46.01%)			84 (48.0%)	81 (41.54%)	77 (49.36%)	242 (46.01%)		
Public servant	14 (7.69%)	22 (7.24%)	3 (7.5%)	39 (7.41%)			12 (6.86%)	21 (10.77%)	6 (3.85%)	39 (7.41%)		
Professionals	13 (7.14%)	27 (8.88%)	2 (5.0%)	42 (7.98%)			10 (5.71%)	14 (7.18%)	18 (11.54%)	42 (7.98%)		
Liberal professions	20 (10.99%)	28 (9.21%)	1 (2.5%)	49 (9.32%)			16 (9.14%)	19 (9.74%)	14 (8.97%)	49 (9.32%)		
Educational level					15.651	0.004					30.927	<0.001
Primary school and below	54 (29.67%)	122 (40.13%)	23 (57.5%)	199 (37.83%)			45 (25.71%)	73 (37.44%)	81 (51.92%)	199 (37.83%)		
Junior middle school	74 (40.66%)	122 (40.13%)	12 (30.0%)	208 (39.54%)			72 (41.14%)	84 (43.08%)	52 (33.33%)	208 (39.54%)		
High school or above	54 (29.67%)	60 (19.74%)	5 (12.5%)	119 (22.62%)			58 (33.14%)	38 (19.49%)	23 (14.74%)	119 (22.62%)		
Smoking					0.383	0.826					0.017	0.992
No	162 (89.01%)	266 (87.5%)	36 (90.0%)	464 (88.21%)			154 (88.0%)	172 (88.21%)	138 (88.46%)	464 (88.21%)		
Yes	20 (10.99%)	38 (12.5%)	4 (10.0%)	62 (11.79%)			21 (12.0%)	23 (11.79%)	18 (11.54%)	62 (11.79%)		
Drinking					2.244	0.326					2.884	0.236
No	176 (96.7%)	290 (95.39%)	40 (100.0%)	506 (96.2%)			170 (97.14%)	184 (94.36%)	152 (97.44%)	506 (96.2%)		
Yes	6 (3.3%)	14 (4.61%)	0 (0.0%)	20 (3.8%)			5 (2.86%)	11 (5.64%)	4 (2.56%)	20 (3.8%)		
Sleep quality					26.995	<0.001					17.015	0.002
Good	101 (55.49%)	133 (43.75%)	14 (35.0%)	248 (47.15%)			93 (53.14%)	97 (49.74%)	58 (37.18%)	248 (47.15%)		
General	68 (37.36%)	150 (49.34%)	15 (37.5%)	233 (44.3%)			75 (42.86%)	83 (42.56%)	75 (48.08%)	233 (44.3%)		
Bad	13 (7.14%)	21 (6.91%)	11 (27.5%)	45 (8.56%)			7 (4.0%)	15 (7.69%)	23 (14.74%)	45 (8.56%)		
Residence					17.467	<0.001					14.350	0.001
Living alone	7 (3.85%)	21 (6.91%)	9 (22.5%)	37 (7.03%)			9 (5.14%)	7 (3.59%)	21 (13.46%)	37 (7.03%)		
Living with other	175 (96.15%)	283 (93.09%)	31 (77.5%)	489 (92.97%)			166 (94.86%)	188 (96.41%)	135 (86.54%)	489 (92.97%)		
Activity					0.730	0.694					26.402	<0.001
Independent activities	174 (95.60%)	287 (94.41%)	37 (92.50%)	498 (94.68%)			174 (99.43%)	188 (96.41%)	136 (87.18%)	498 (94.68%)		
Assistance needed	8 (4.40%)	17 (5.59%)	3 (7.50%)	28 (5.32%)			1 (0.57%)	7 (3.59%)	20 (12.82%)	28 (5.32%)		
Payment					0.208	0.901					0.853	0.653
Medical insurance	160 (87.91%)	266 (87.5%)	36 (90.0%)	462 (87.83%)			156 (89.14%)	168 (86.15%)	138 (88.46%)	462 (87.83%)		
Self paid	22 (12.09%)	38 (12.5%)	4 (10.0%)	64 (12.17%)			19 (10.86%)	27 (13.85%)	18 (11.54%)	64 (12.17%)		
Medication history					8.823	0.012					13.072	0.001
No	53 (29.12%)	56 (18.42%)	6 (15.0%)	115 (21.86%)			54 (30.86%)	37 (18.97%)	24 (15.38%)	115 (21.86%)		
Yes	129 (70.88%)	248 (81.58%)	34 (85.0%)	411 (78.14%)			121 (69.14%)	158 (81.03%)	132 (84.62%)	411 (78.14%)		
Surgical history					2.793	0.247					25.250	<0.001
No	71 (39.01%)	96 (31.58%)	14 (35.00%)	181 (34.41%)			86 (49.14%)	52 (26.67%)	43 (27.56%)	181 (34.41%)		
Yes	111 (60.99%)	208 (68.42%)	26 (65.00%)	345 (65.59%)			89 (50.86%)	143 (73.33%)	113 (72.44%)	345 (65.59%)		
Number of underlying diseases					18.235	0.006					37.830	<0.001
0	32 (17.58%)	34 (11.18%)	2 (5.0%)	68 (12.93%)			36 (20.57%)	15 (7.69%)	17 (10.9%)	68 (12.93%)		
1	83 (45.6%)	111 (36.51%)	17 (42.5%)	211 (40.11%)			80 (45.71%)	81 (41.54%)	50 (32.05%)	211 (40.11%)		
2	59 (32.42%)	120 (39.47%)	16 (40.0%)	195 (37.07%)			46 (26.29%)	87 (44.62%)	62 (39.74%)	195 (37.07%)		
3 or more	8 (4.4%)	39 (12.83%)	5 (12.5%)	52 (9.89%)			13 (7.43%)	12 (6.15%)	27 (17.31%)	52 (9.89%)		
Self-care ability					3.430	0.753					5.646	0.464
Complete self-help	21 (11.54%)	37 (12.17%)	3 (7.5%)	61 (11.6%)			15 (8.57%)	23 (11.79%)	23 (14.74%)	61 (11.6%)		
Mild dependence	140 (76.92%)	223 (73.36%)	33 (82.5%)	396 (75.29%)			137 (78.29%)	145 (74.36%)	114 (73.08%)	396 (75.29%)		
Moderate dependence	12 (6.59%)	21 (6.91%)	3 (7.5%)	36 (6.84%)			13 (7.43%)	16 (8.21%)	7 (4.49%)	36 (6.84%)		
Severe dependence	9 (4.95%)	23 (7.57%)	1 (2.5%)	33 (6.27%)			10 (5.71%)	11 (5.64%)	12 (7.69%)	33 (6.27%)		
Hearing					0.373	0.830					22.375	<0.001
Normal	138 (75.82%)	230 (75.66%)	32 (80.00%)	400 (76.05%)			154 (88.00%)	142 (72.82%)	104 (66.67%)	400 (76.05%)		
Abnormal	44 (24.18%)	74 (24.34%)	8 (20.00%)	126 (23.95%)			21 (12.00%)	53 (27.18%)	52 (33.33%)	126 (23.95%)		
Visual impairment					26.765	<0.001					22.375	<0.001
Mild	69 (37.91%)	78 (25.66%)	9 (22.5%)	156 (29.66%)			69 (39.43%)	54 (27.69%)	33 (21.15%)	156 (29.66%)		
Middle	102 (56.04%)	211 (69.41%)	22 (55.0%)	335 (63.69%)			95 (54.29%)	131 (67.18%)	109 (69.87%)	335 (63.69%)		
Severe	11 (6.04%)	15 (4.93%)	9 (22.5%)	35 (6.65%)			11 (6.29%)	10 (5.13%)	14 (8.97%)	35 (6.65%)		
Falls history in the past 6 months					149.204	<0.001					22.743	<0.001
No	182 (100.00%)	304 (100.00%)	28 (70.00%)	514 (97.72%)			175 (100.00%)	194 (99.49%)	145 (92.95%)	514 (97.72%)		
Yes	0 (0.00%)	0 (0.00%)	12 (30.00%)	12 (2.28%)			0 (0.00%)	1 (0.51%)	11 (7.05%)	12 (2.28%)		
Height	161.42 ± 7.04	161.92 ± 7.21	161.80 ± 6.99	161.74 ± 7.13	0.281	0.755	161.61 ± 7.08	161.80 ± 7.32	161.80 ± 6.97	161.74 ± 7.13	0.043	0.957
Weight	62.99 ± 11.61	64.02 ± 11.91	61.00 ± 10.34	63.43 ± 11.71	1.373	0.254	63.83 ± 11.86	63.28 ± 11.61	63.19 ± 11.71	63.43 ± 11.71	0.149	0.862

Chi-square test was used for categorical variable comparisons; Fisher’s exact test was used for small cell counts. Kruskal-Wallis H test was used for continuous or ranked variables. Dunn’s *Post-hoc* test with Benjamini-Hochberg correction was used for pairwise comparisons among groups with significant Kruskal-Wallis H test (*P* < 0.05).

### 3.3 Fall awareness level

The total score of the fall awareness Scale for 526 patients was 54.45, indicating a moderate level. The four subscales of the SAFE scale comprised 21 items in total (8 items for activity safety and environmental alertness, 7 items for physical function alertness, 4 items for medication alertness, and 2 items for cognitive-behavioral alertness). Using the tertile of the total score as the cutoff, fall awareness was categorized into low level (≤51 points), medium level (52–57 points), and high level (≥58 points), with 175 cases (33.27%), 195 cases (37.07%), and 156 cases (29.66%) respectively. Univariate analysis showed statistically significant differences (*p* < 0.05) among patients with different fall awareness levels in terms of age, marital status, education level, sleep quality, living arrangement, activity pattern, medication history, surgical history, number of underlying diseases, vision, hearing, and fall history within the past 6 months. *Post-hoc* Dunn-BH pairwise comparisons further indicated significant differences in fall awareness levels across subgroups of age, marital status, education level, sleep quality, living arrangement, activity pattern, medication history, surgical history, number of underlying diseases, vision, hearing, and fall history within the past 6 months (all *p* < 0.05), shown in Table 2.

### 3.4 Inter-group differences in fall awareness among hospitalized elderly cataract patients with different fall risk levels

Patients in the low-risk fall group exhibited a fall awareness score of 55.00 (49.75, 59.00); those in the medium-risk fall group scored 54.00 (49.0, 58.00); and patients in the high-risk fall group scored 55.00 (52.00, 60.00). The result indicating that fall awareness levels did not differ significantly across different JHFRAT risk strata (H = 1.450, *p* > 0.05).

### 3.5 Intra-group differences in fall awareness among hospitalized elderly patients with cataracts at different fall risk levels

**Figure 1:**
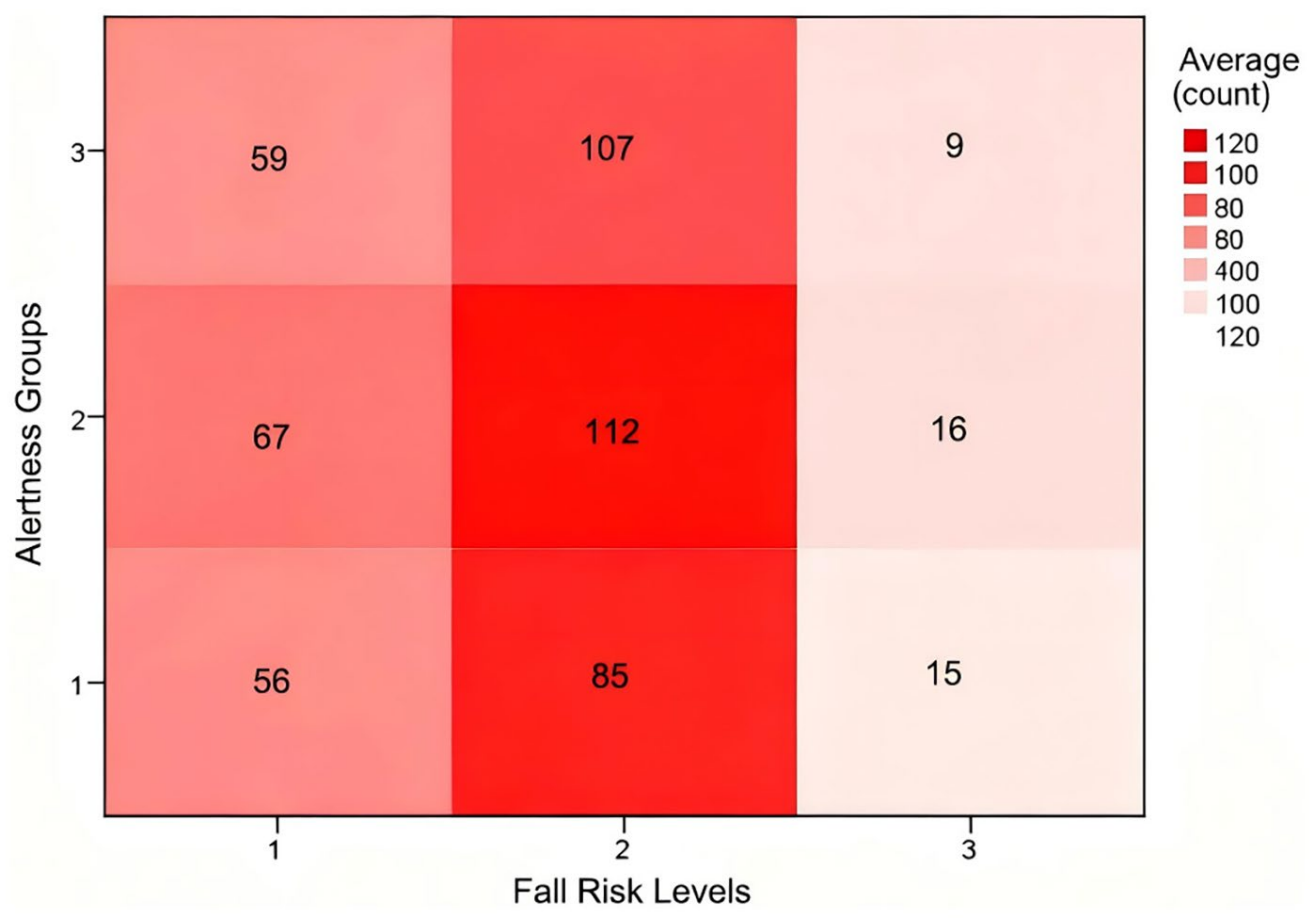
Heatmap of case distribution across fall risk levels and alertness groups.

To describe the distribution of fall awareness deviation among patients with different fall risk levels, the distribution of fall awareness levels was compared among the low-risk, intermediate-risk, and high-risk groups. The case distribution across fall risk levels and alertness groups is presented in Figure 1. In terms of distribution characteristics: The low-risk fall group (*n =* 182) exhibited 59 cases of excessively low alertness (32.42%), 67 cases of moderate alertness (36.81%), and 56 cases of excessively high alertness (30.77%), with a predominant pattern of excessive alertness. The intermediate-risk fall group (*n =* 304) included 107 cases of excessively low alertness (35.20%), 112 cases of moderate alertness (36.84%), and 85 cases of excessively high alertness (27.96%), with a distribution pattern characterized by coexisting insufficient and excessive alertness. The high-risk fall group (*n =* 40) showed 9 cases of excessively low alertness (22.50%), 16 cases of moderate alertness (40.00%), and 15 cases of excessively high alertness (37.50%), with an predominant pattern of insufficient alertness. (Table 3).

**Table 3:** Comparative analysis of fall risk alertness among hospitalized elderly cataract patients with different fall risk levels.

Fall risk stratification	Fall risk alertness grouping	Number [*n* (%)]	Fall risk score [Q50(Q25, Q75)]	Insufficient alertness [*n* (%)]	Excessive alertness [*n* (%)]	*χ* ^2^	*p*-value
Low-risk (*n =* 182)	Low level	59 (32.42)	46.00 (42.00, 49.00)	–	123 (67.58)	3.074	0.545
	Medium level	67 (36.81)	55.00 (53.00, 56.00)				
	High level	56 (30.77)	65.50 (59.00, 71.00)				
Medium-risk (*n =* 304)	Low level	107 (35.20)	46.00 (41.00, 49.00)	107 (35.20)	85 (27.87)		
	Medium level	112 (36.84)	55.00 (53.00, 56.00)				
	High level	85 (27.96)	66.00 (59.00, 71.50)				
High-risk (*n =* 40)	Low level	9 (22.50)	40.00 (37.50, 47.50)	25 (62.50)	–		
	Medium level	16 (40.00)	53.50 (52.25, 55.00)				
	High level	15 (37.50)	60.00 (59.00, 70.00)				

## 4 Discussion

### 4.1 The fall risk and fall awareness in elderly hospitalized cataract patients are both at medium-to-high levels

The results of this study indicate that among hospitalized elderly patients with cataracts, the majority (57.79%) exhibited moderate fall risk, while high-risk individuals accounted for 7.60%. The Johns Hopkins Fall Risk Assessment Tool was employed to evaluate patients’ fall risk across seven dimensions: age, fall history within the past 6 months, excretion patterns, medication use, urinary catheter status, physical activity, and cognitive function. Previous studies have demonstrated a strong correlation between age and fall incidence (19). In this study, patients aged ≥70 years accounted for 52.85%. As age advances, the regulatory capacity of the central nervous system declines, resulting in diminished sensory functions, including vision, touch, and hearing, as well as concurrent degenerative changes in joints and bones. This makes individuals prone to gait instability, thus increasing the risk of falls. Additionally, comorbid underlying diseases and the use of medications are associated with falls, and these findings are consistent with those reported by Qiu et al. (20). And 87.07% of patients had ≥1 underlying disease, which may increase the risk of falls by impairing motor coordination, balance, and stability or prolonging neural reflex time. Additionally, 78.14% of patients required long - term oral administration of multiple medications (e.g., antihypertensive drugs, hypoglycemic agents), which may induce adverse reactions such as orthostatic hypotension and dizziness, thereby further increasing the risk of falls. Sun (21) study revealed that visual impairment is a significant risk factor for falls in the elderly. In this study, 70.34% of patients had visual acuity below 0.3. Insufficient visual input can impair balance control and obstacle avoidance abilities, leading to distance judgment errors and restricted spatial information processing. These factors reduce patients’ independence in daily activities, thereby increasing their susceptibility to falls. Additionally, visual impairment may adversely affect mental health in older adults, heightening fall-related anxiety—a critical risk factor for falls. This psychological impact may partially explain the elevated fall risk observed among the study subjects.

The total fall awareness score among hospitalized elderly cataract patients was 54.46, generally at a moderate level. This indicates that the alertness levels in this population still need improvement and remain below the actual fall risk level. Dimension scores of Fall Awareness Scale were as follows: activity safety and environmental alertness (20.10 points), physical function alertness (17.40 points), medication alertness (8.86 points), and cognitive - behavioral alertness (8.10 points).

It shows the activity safety and environmental alertness scores were relatively high, which is consistent with the findings of Hu (17). This suggests that patients have certain vigilance and recognition capabilities regarding fall risks in their surroundings. In contrast, the medication alertness scores were lower, in line with Liu et al.’s study (22). Potential reasons include some elderly patients’ insufficient awareness of adverse drug reactions (e.g., orthostatic hypotension caused by antihypertensive medications). They tend to attribute related symptoms to comorbidities or age- related factors rather than recognize drug-related risks. This may be associated with the drug tolerance developed through long-term medication use and inadequate risk education. Clinicians should strengthen medication safety education for hospitalized elderly cataract patients and their families to help establish accurate associations between medications and fall risks, thereby improving adherence to preventive behaviors. Additionally, the cognitive-behavioral alertness scores were the lowest in this study, consistent with Wei et al.’s findings (23). Some elderly individuals, influenced by innate cognitive patterns and behavioral habits, may prefer self-management and avoid seeking assistance. This may lead to overly optimistic assessments of their mobility or insufficient attention to risk scenarios. During hospitalization, healthcare professionals should conduct targeted risk communication and behavioral guidance based on the patient’s functional status, enhance their rational understanding of their own mobility capabilities, and reduce falls caused by overconfidence or risk neglect.

### 4.2 The deviation in fall awareness among elderly hospitalized cataract patients

This study found no statistically significant difference in fall awareness scores among patients with different fall risk levels (H = 1.450, *p* > 0.05), indicating no linear correlation between objective risk stratification and subjective alertness levels. These results confirm the necessity of fall awareness as an independent psychological dimension. Objective assessments reflect risk exposure levels from healthcare providers’ perspectives, while subjective alertness is embedded in patients’ disease experiences, functional compensation states, and health beliefs. Their distinct information sources and formation pathways lead to “dual-track parallelism” in clinical manifestations. Therefore, relying solely on traditional risk assessment scales for fall management may overlook patients’ risk perception biases, particularly failing to detect high-risk psychological states such as “blind confidence” and “excessive fear.”

The study further reveals that deviations between objective risk and subjective alertness follow systematic patterns rather than random distribution. Among high- risk fall groups, 62.50% exhibited insufficient alertness; medium-risk groups showed 35.20% with insufficient alertness and 27.96% with excessive alertness; low-risk groups had 67.58% with excessive alertness. These findings align with Delbaere et al.’s (24) community-based elderly studies and Lim et al.’s (25) hospitalized elderly populations, suggesting that “alertness-risk mismatch” is prevalent across elderly populations but varies in incidence rates across studies. Delbaere et al. (24) reported an under-alertness rate of 20%, lower than the 67.6% observed in this study; Lim et al. (25) documented an over-alertness rate of 50.7%, also below the 64.7% recorded in our research. These discrepancies may stem from differences in study settings and sample characteristics. On one hand, our study subjects were hospitalized elderly patients whose health status and functional levels fluctuated during treatment, potentially altering alertness levels. Some elderly patients with multiple comorbidities exhibited high objective risks but failed to maintain heightened alertness due to “risk habituation” from prolonged illness or inadequate awareness of fall consequences. In contrast, community - dwelling seniors generally maintained stable health conditions, likely resulting in higher under - alertness rates among hospitalized populations. On the other hand, our participants had a mean age of 68.87 years (compared to Lim et al.’s 75.30 years), and prior studies indicate age - related variations in risk perception (26). Younger patients with better self-care abilities may develop generalized fear through indirect experiences (e.g., witnessing falls), maintaining excessive alertness despite low objective risks—a phenomenon that partially explains the higher over-alertness rate observed in this study. Under- alertness directly increases fall risk, while over - alertness, though beneficial for short -term risk avoidance, may lead to activity avoidance, sarcopenia, frailty, and declining quality of life over time, creating a vicious cycle of “fear→immobilization→ functional decline→elevated fall risk.”Therefore, clinical practice should incorporate simultaneous assessments based on objective risk stratification.

### 4.3 Differences in influencing factors of fall risk and fall awareness

Univariate analysis revealed that the common risk factors for fall risk and fall awareness included age, marital status, education level, sleep quality, residence, number of underlying diseases, visual impairment, and fall history within the past 6 months. These factors serve not only as objective risk predictors but also contribute to patients’ subjective risk perception, and should be included as baseline items in fall prevention screening. The differential variables between the two groups included activity patterns, surgical history, and hearing impairment, which were significantly associated only with fall awareness (*p* < 0.001) and showed no statistically significant correlation with objective fall risk. This finding offers crucial insights into the mechanisms underlying vigilance formation. In our study, participants in assisted activities displayed significantly higher vigilance levels than those in independent activities, which is consistent with the conclusions of a community - based elderly study (27). This might be due to the decline in physical function leading to excessive anxiety related to falls, which reduces physical activity and, in turn, increases the risk of falls. A systematic review demonstrated that elderly individuals who regularly engage in exercises like Tai Chi and balance training not only improve their physical function but also significantly reduce their fear of falling and enhance their confidence in self-control, thus adjusting vigilance to an optimal level (28). Patients with a surgical history showed significantly higher vigilance than those without, especially among those who had fall-related surgeries (e.g., post - hip fracture surgery). A follow - up study on total knee arthroplasty patients found that over 40% of patients still had a pronounced “fear of falling” 6 months after the operation, which did not fully match the actual physical functional recovery (29). Although surgical history was not specifically classified as a fracture - related factor in our study, a significant association was observed, suggesting potential connections to role - reinforcement effects from surgical experiences and repeated fall prevention education during rehabilitation. Notably, some post-cataract surgery patients developed a “recovery achieved” cognitive bias because of improved vision, potentially resulting in decreased vigilance. This emphasizes the importance of subgroup analysis considering the type of surgery and the postoperative duration. Hearing - impaired individuals exhibited significantly higher vigilance than those with normal hearing, although hearing status showed no significant correlation with the objective fall risk in this study. The impact of hearing loss on fall vigilance may be underestimated. Patients with hearing loss often face a heightened fall risk due to impaired perception of environmental sounds and reduced balance capacity, which heightens their awareness of potential hazards. Studies indicate that elderly individuals with mild hearing loss who receive no intervention may experience nearly three times the incidence of falls compared to their peers (30, 31). Furthermore, research highlights the critical role of audiologists in fall prevention among the elderly, recommending the inclusion of hearing screenings and fall fear assessments during hospital admission evaluations for senior patients (32).

### 4.4 Developing fall prevention management strategies by integrating fall assessment and patient fall awareness

This study uncovered a widespread cognitive bias between objective fall risk and subjective fall awareness among hospitalized elderly cataract patients, with disparities in bias patterns varying across different risk levels. However, current clinical fall management practices still predominantly rely on objective risk stratification as the core basis for interventions. For example, warning signs are uniformly displayed, and the assessment frequency is increased for high-risk patients, while low-risk and intermediate-risk patients only receive verbal education. Although this tiered intervention model is easy to implement, it has the following limitations: ① It fails to identify “insufficient alertness” in high - risk groups, who are in the greatest need of cognitive arousal but may be overlooked due to a lack of expressed concerns; ② It is unable to address “excessive alertness” in low - risk groups, who most require psychological counseling and activity guidance but are classified as routine education cases because of their low risk levels; ③ It fails to activate patients’ intrinsic motivation as active participants in fall prevention. Fall prevention is not merely a nursing task but a behavioral adjustment process that requires patient participation. Delbaere et al. (24) emphasized that fall risk assessment should encompass both objective risk evaluation and perceived risk assessment. The study suggests incorporating a fall awareness scale into the routine nursing assessments of hospitalized elderly patients, completing dual - scale screening for fall risk and alertness within 24 h of admission, and identifying high - risk individuals with fall awareness scores ≤51 (low alertness) as key warning targets. Additionally, different health education approaches can more precisely enhance patients’ fall awareness levels (33). Precision health education should be implemented for patients with low fall risk awareness. For example, specialized lectures on “Medication and Falls” should be conducted for individuals with low drug alertness, focusing on the dosing schedules of antihypertensive agents, sedative - hypnotics, and hypoglycemic drugs, as well as adverse reaction identification and activity safety. For patients with low cognitive behavioral alertness, motivational interviews should be used to correct rationalization beliefs such as “occasional incidents are harmless” or “slowing down is sufficient,” thereby helping patients establish risk perceptions in line with their functional status.

## 5 Limitation

This study has several limitations. First, this was a cross-sectional study, which can only demonstrate associations rather than causal relationships between fall risk and fall awareness. Second, the study was conducted at a single center with convenience sampling, which may limit the generalizability of the findings to community-dwelling or primary-care cataract patients. Third, in terms of variable measurement, surgical history was analyzed as a binary variable and not further stratified by type (cataract vs. orthopedic/other surgery), laterality, or postoperative duration; hearing was assessed by self-report rather than objective audiometry; and visual acuity measurement lacked full standardization across examiners, lighting, and distance, which may introduce bias. Fourth, we did not follow up post-discharge falls, so the predictive validity of awareness mismatch for actual fall events remains unclear. Fifth, the Hawthorne effect may have temporarily increased patients’ alertness during assessment, potentially influencing SAFE scores.

## 6 Conclusion

This study conducted a cross-sectional survey of 526 hospitalized elderly cataract patients to systematically evaluate the current status, distribution characteristics, and influencing factors of fall risk and fall awareness. The novelty and uniqueness of this study is confirming the specific risk-awareness mismatch pattern: insufficient alertness in high-risk patients and excessive alertness in low-risk patients among elderly cataract inpatients. The fall risk among hospitalized elderly cataract patients was at a moderate level (57.79% of cases classified as moderate risk), with a total fall awareness score of 54.45 points, also indicating a moderate level. However, no statistically significant differences in fall awareness scores were observed across different fall risk levels, suggesting a lack of consistency between objective risk stratification and subjective alertness levels. Traditional fall risk assessment tools struggle to capture patients’ subjective perception of their own risk, and the two cannot be substituted for each other. A pervasive perceptual bias exists between fall risk and fall awareness, with the direction of bias showing a pattern-dependent distribution across risk levels. In high-risk fall groups, insufficient alertness predominated (62.50%), while low-risk groups exhibited excessive alertness (67.58%). Medium-risk fall groups displayed a polarized pattern of both insufficient and excessive alertness. This indicates that the “risk-alertness mismatch” phenomenon is prevalent among hospitalized elderly cataract patients, with deviation types showing significant correlations with risk levels. The influencing factors for fall risk and fall awareness overlap but also exhibit significant differences. Age, marital status, education level, sleep quality, housing patterns, underlying diseases, visual acuity, and a history of falls within the past 6 months were common factors affecting both. In contrast, activity patterns, surgical history, and hearing status were only significantly associated with fall awareness, showing no statistical correlation with objective fall risk. The findings reveal a unique formation mechanism of fall awareness, which is not only driven by objective risk exposure but also regulated by functional compensation status, disease experience, and multisensory reserves. This study demonstrates the significant clinical value of incorporating fall awareness assessment into routine nursing screening for hospitalized elderly patients. Based on the mismatch characteristics between fall risk and alertness, a four-type cognitive classification framework (blind confidence type, excessive fear type, cognitive matching type, and contradictory perception type) may be constructed in future research to support targeted stratified intervention design. By integrating patients’ subjective cognitive dimensions and optimizing the fall risk assessment system, the precision of fall prevention interventions and patient engagement can be enhanced, thereby effectively reducing the incidence of falls among hospitalized elderly cataract patients.

## Data Availability

The raw data supporting the conclusions of this article will be made available by the authors, without undue reservation.
